# The retinal function imager and clinical applications

**DOI:** 10.1186/s40662-018-0114-1

**Published:** 2018-08-12

**Authors:** Daniel Su, Sunir Garg

**Affiliations:** The Retina Service of Wills Eye Hospital, Mid Atlantic Retina, 840 Walnut Street, Suite 1020, Philadelphia, PA 19107 USA

**Keywords:** Retinal function imager, Retinal blood flow, Blood flow velocity, Oximetry, Noninvasive imaging, Retina

## Abstract

**Background:**

The Retinal Function Imager (RFI) provides in vivo and noninvasive imaging of both the retinal structure and function.

**Review:**

The RFI can create capillary perfusion maps, measure blood flow velocity, and determine metabolic function including blood oximetry. It can aid clinical diagnosis as well as assess treatment response in several retinal vascular diseases including diabetic retinopathy. Blood flow velocity abnormalities have also been implicated in disease such as age-related macular degeneration and require further investigation. Compared with optical coherence tomography angiography, the RFI produces capillary maps of comparable image quality and wider field of view but it is unable to provide depth-resolved information and has longer image acquisition time. Currently, functional imaging using blood oximetry has limited applications and additional research is required.

**Conclusion:**

The RFI offers noninvasive, high-resolution imaging of retinal microvasculature by creating capillary perfusion maps. In addition, it is capable of measuring retinal blood velocity directly and performs functional imaging with retinal blood oximetry. Its clinical applications are broad and additional research with functional imaging may potentially lead to diagnosis of diseases and their progression before anatomic abnormalities become evident, but longer image acquisition times may limit its clinical adoption.

## Background

A wide spectrum of ophthalmic diseases, including those of the retina, have been described and studied through direct visualization. This ability has encouraged the development of innovative imaging technologies. Intravenous fluorescein angiography (IVFA) has been the gold standard for studying and diagnosing retinal vascular abnormalities and is readily available [1]. However, it requires an intravenous injection of a dye, which carries a small but not insignificant risk of adverse reactions ranging from nausea to anaphylaxis [2, 3]. In addition, IVFA is relatively contraindicated in pregnancy and obtaining intravenous access can be difficult and time-consuming. These limitations, along with advances in computational technology, have spurred development of non-invasive, real-time imaging modalities. The Retinal Function Imager (RFI) (Optical Imaging Ltd., Rehovot, Israel) provides in vivo and noninvasive imaging of both retinal structure and function. It can create capillary perfusion maps (noninvasive angiography), directly measure blood flow velocity, and determine metabolic function including blood oximetry [4]. This paper aims to review the RFI platform and its clinical applications.

### Main text

#### Technical specifications

The RFI system is composed of a fundus camera, stroboscopic illumination, fast filter wheel, and LED-based stimulus generator [5]. Fast stroboscopic illumination enables the camera to take multiple snapshots of the retina in less than 0.2 s. This high speed is required in order to reduce inter-frame retinal motion and to track the movement of red blood cells (RBCs) through each sequential frame [6]. Using multiple sequences, it creates capillary perfusion maps and performs blood flow velocity measurements. The fast filter wheel is capable of rapidly switching up to four different illumination wavelengths, allowing for multiple wavelength image acquisition with minimal eye movement. A qualitative blood oximetry map can be produced from different reflections of the retinal vasculature using varying wavelengths. Lastly, retinal reflectance changes in response to the LED-based stimulus generator carry information about metabolic processes that are useful for functional signal imaging.

In addition to these functions, the RFI system also includes standard color fundus photography, fluorescein angiography, indocyanine green angiography, and autofluorescence.

#### Capillary perfusion map

By directly tracking the movement of RBCs, the RFI is able to create a map of the retinal microvasculature. When studying retinal microvasculature visualized with IVFA compared to RFI, Witkin et al. found that RFI could visualize a higher order of vessel branching. In addition, the foveal avascular zone (FAZ) was more clearly delineated and appears smaller when using RFI [7]. This was partly due to the resolution obtained by imaging individual RBCs. In addition, in IVFA the retinal vasculature could become difficult to differentiate from choroidal hyperfluorescence.

Detection of vessel abnormalities is paramount in the diagnosis of diabetic retinopathy. Early vessel dysfunction, as demonstrated by increased vascular shunts and FAZ enlargement may be accurately assessed with RFI [8]. Neovascularization over the optic nerve in proliferative diabetic retinopathy can also be visualized with RFI [8].

**Fig. 1:**
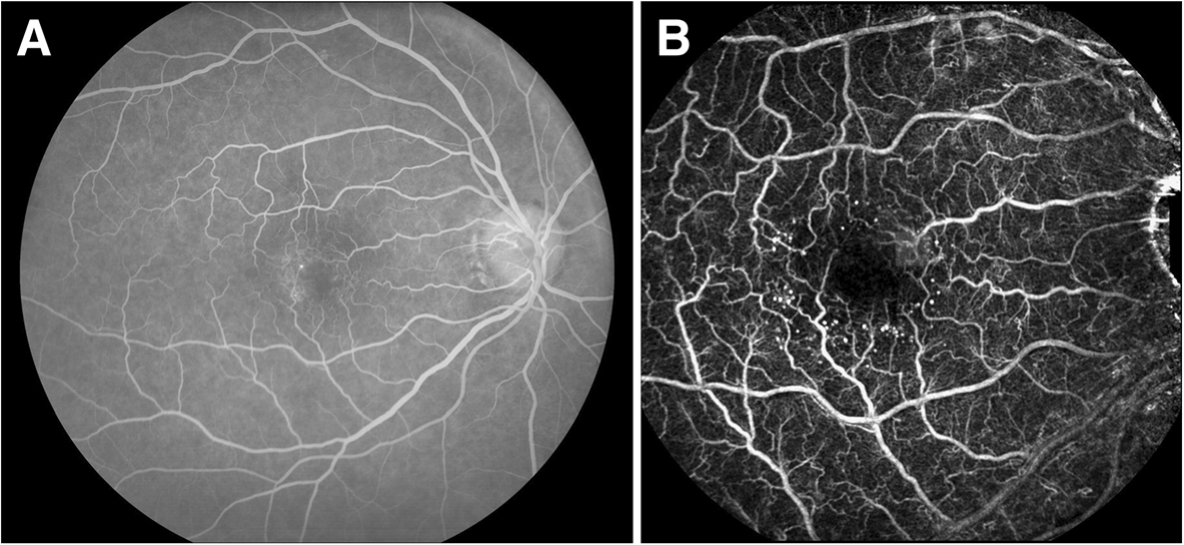
Retinal function imager capillary perfusion map compared to fluorescein angiogram. **A** The fluorescein angiogram (FA) demonstrates a small microaneurysm as a focal spot of hyper-fluorescence. **B** In comparison, the RFI capillary perfusion map (**B**) reveals multiple small microaneurysms and provides a higher level of detail of the capillary vessels. FA may demonstrate increased vascular permeability as dye leakage but this cannot be ascertained in a single snapshot

The main advantage of RFI over IVFA is its noninvasive nature, which can be helpful in cases of difficult vascular access or prior adverse reaction. In addition, RFI provides more detailed visualization of the retinal vasculature, revealing capillary vessels and vessel characteristics such as vessel loops and vertical collateral vessels more easily than IVFA (Fig. 1). On the other hand, increased vascular permeability that is readily revealed by fluorescein leakage in cases such as macular edema and optic disk edema, cannot be demonstrated with RFI or other noninvasive imaging modalities.

#### Blood flow velocity

The 1024 × 1024 pixel digital camera and stroboscopic flash enable the RFI system to capture a series of 8 images at 50–60 Hz in less than 0.2 s. Under green illumination, hemoglobin within the RBCs provides a natural, high-contrast chromophore, allowing the RFI to track the movement of individual RBCs through the 8 sequential images. The direct measurement of the distance traveled by the RBCs divided by the time it takes to capture the series of images yields a direct measure of blood flow velocity. Image acquisition is synchronized to the cardiac cycle (through a probe attached to the fingertip or earlobe) to control for the effect of arteriolar pulsations. Segmental blood flow velocity measurements have been shown to be reproducible [9]. Conversion from a velocity map to a flow map requires precise measurement of vessel diameter across each flow segment, which can be time-consuming with manual measurements of each individual vessel diameter. However, recent software can automatically identify and measure all vessel segments, greatly simplifying blood flow measurements [10].

Retinal blood flow abnormalities may be seen in diseases such as hypertensive retinopathy (vessel narrowing), diabetic retinopathy, retinal vein occlusion and arteriole occlusion. Burgansky-Eliash et al. demonstrated significantly decreased flow velocities in the retinal arterioles and venules of patients with nonproliferative diabetic retinopathy compared with healthy controls [11]. Furthermore, a more recent study showed changes in flow velocity in diabetic patients but without visible retinopathy compared with healthy controls, suggesting detectable physiological changes prior to the development of anatomic abnormalities [12]. The ability to consistently measure blood flow over specific vessel fragments may facilitate earlier diagnosis and for following treatment response. In addition, retinal blood flow velocity has been shown to be correlated with coronary blood flow, hypertension, and systemic metabolic syndrome, and therefore it may be a screening tool or prognosticator for systemic cardiovascular morbidity [13–15].

Recent clinical studies utilizing RFI in age-related macular degeneration (AMD) demonstrated reduced blood-flow velocities compared with healthy controls [16]. Additional studies measured retinal blood flow following anti-vascular endothelial growth factor treatment in eyes with neovascular AMD and found an increase in retinal blood flow that correlated with an improvement in visual acuity [17, 18]. Further studies are required to investigate the role of retinal blood flow and its role in AMD.

#### Retinal oximetry

The retinal photoreceptors have the highest metabolic demand of any tissue in the body. Alterations in oxygen supply or demand may indicate early onset of retinal abnormalities [4]. In multispectral imaging mode, the RFI can detect differences between the absorption spectra of oxyhemoglobin and deoxyhemoglobin in order to determine the oxygenation of blood. However, the accuracy of retinal oximetry is limited by variations in retinal pigmentation and the result may be difficult to interpret [19]. Nevertheless, newer algorithms are being developed to overcome this limitation [10]. Accurate oxygen saturation maps may provide additional details in the pathophysiology of various retinal diseases.

#### Functional imaging

Using near-infrared light (750–840 nm), the RFI is able to image the retina outside of the absorption range of photoreceptors. The difference between the pre- and post-stimulated images in response to a well-defined visual stimulus such as a light is used to determine the metabolic state of the retina. This feature is experimental with limited clinical applications. However, future improvements in functional imaging may provide additional insights into disease pathology.

#### Optical coherence tomography angiography

**Fig. 2:**
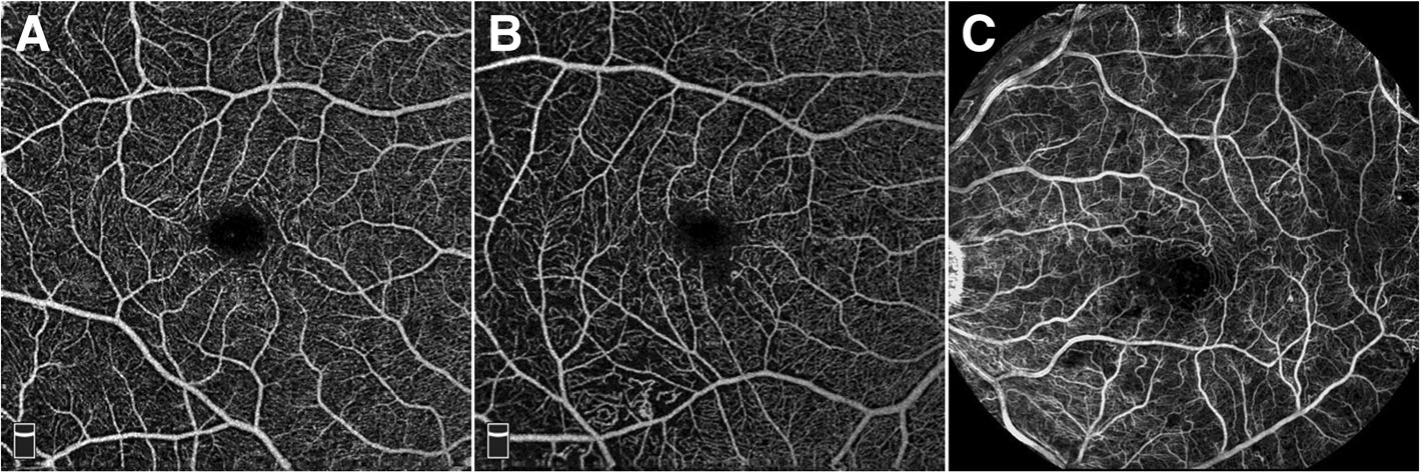
Retinal function imager capillary perfusion map compared to optical coherence tomography angiography. **A**. Optical coherence tomography angiography (OCTA) of a normal eye. **B**. OCTA of an eye with diabetic retinopathy with an irregular foveal avascular zone as well as capillary dropout in the inferotemporal macula. **C**. Capillary perfusion map imaged using the Retina Function Imager (RFI) showing a wider field of view with greater capillary details

Also noninvasive, optical coherence tomography angiography (OCTA) is a relatively new imaging modality that generates 3-dimensional, depth-encoded images of blood flow within the eye by motion contrast. Using rapid OCT scanning, multiple A-scans acquired at the same location in the retina are compared to detect motion from blood flow [20]. In addition to providing detailed maps of the retinal vasculature, OCTA is able to provide depth-resolved information that can be used to isolate vascular structures in different layers of the retina and visualize them individually. Moreover, the noninvasive angiography images are cross-registered with structural OCT B-scans for precise co-localization of pathology (Fig. 2).

**Fig. 3:**
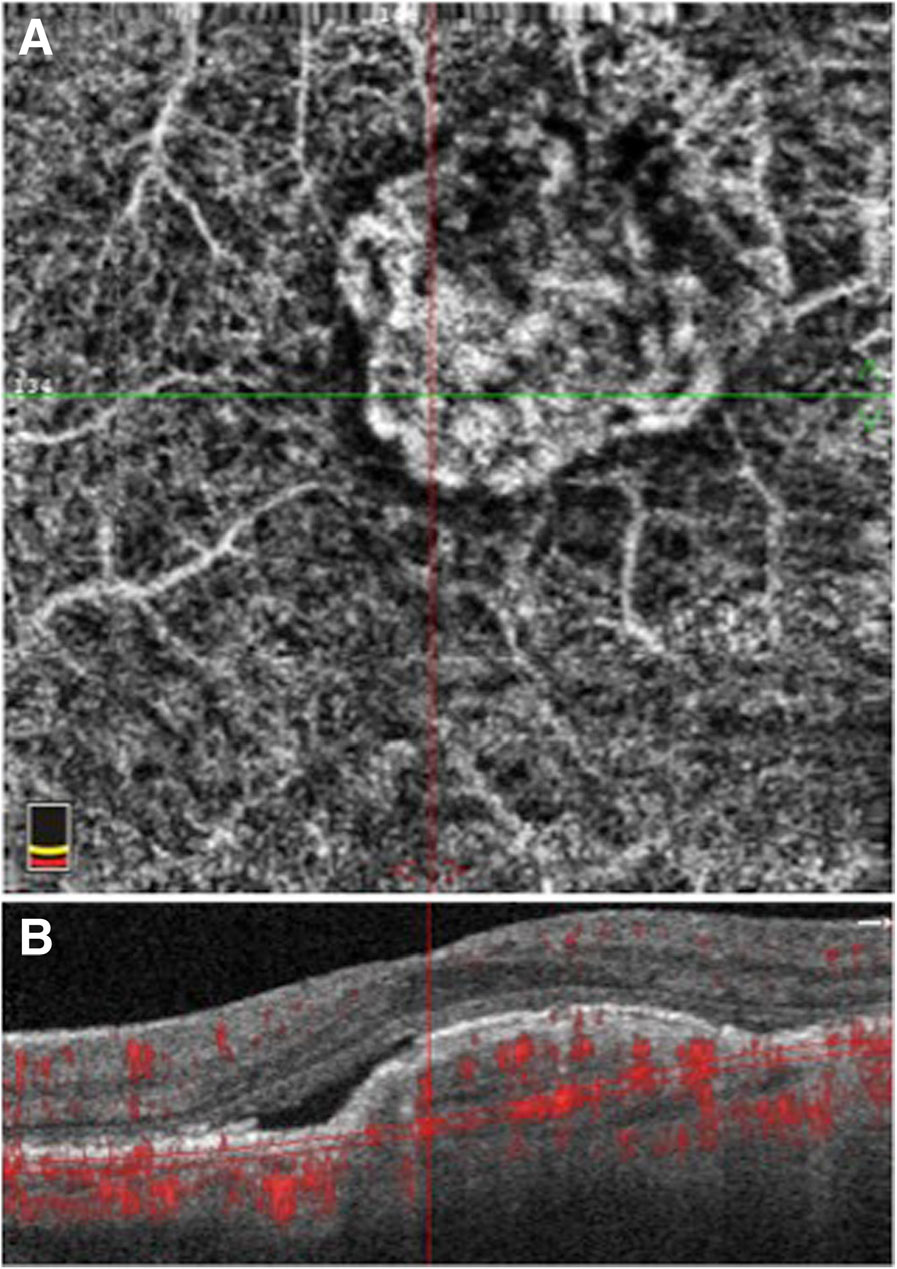
Projection artifacts in optical coherence tomography angiography. **A**. *En face* OCTA of an eye containing a neovascular lesion. OCTA was able to segment specific retinal layers and display them individually. However, a major limitation is the projection artifacts shown here, specifically the superficial retinal vessels that are displayed in a segmented slab of the choriocapillaris. **B**. Corresponding structural OCT B-scan demonstrating the neovascular lesion co-localized to the *en face* image

Compared with commercially available OCTA systems, RFI is able to produce a capillary map with a larger field of view (up to 7.4 × 7.4 mm^2^ compared to 3 × 3 mm^2^ for OCTA), which covers larger areas of clinical interest and with a higher pixel count. This may produce images with closer details of the vascular network (Fig. 3). In addition, the RFI is able to measure blood flow velocity directly and can identify potential functional irregularities by measuring metabolic activity. However, unlike depth-encoded images from OCTA, the RFI is unable to differentiate the depth between different vascular networks due to the nature of *en face* imaging. In addition, OCTA technology continues to evolve rapidly. For example, swept source widefield OCTA that is currently under development is able to obtain 12 mm × 6 mm OCTA images in 4 s [21].

## Conclusions

In addition to traditional fundus photography and IVFA, the RFI enables noninvasive, high-resolution imaging of retinal microvasculature by creating capillary perfusion maps. In addition, it is capable of directly measuring retinal blood velocity and perform functional imaging with retinal blood oximetry. Detection of structural abnormalities through capillary perfusion maps is clinically important but not unique to the RFI. While commercially available OCTA platforms can also identify vascular structural abnormalities, the RFI is unique in its ability to potentially detect functional abnormalities. This may lead to diagnosis of diseases and their progression before anatomic abnormalities become evident, allowing for earlier intervention. In addition, functional imaging may open up research and therapeutic development opportunities involving a wide spectrum of retinal diseases, potentially leading to improved outcomes.

## Abbreviations

AMD: Age-related macular degeneration
FAZ: Foveal avascular zone
IVFA: Intravenous fluorescein angiography
OCTA: Optical coherence tomography angiography
RBCs: Red blood cells
RFI: Retinal Function Imager

## References

[1] Brancato R, Trabucchi G (1998). Fluorescein and indocyanine green angiography in vascular chorioretinal diseases. Semin Ophthalmol.

[2] Yannuzzi LA, Rohrer KT, Tindel LJ, Sobel RS, Costanza MA, Shields W (1986). Fluorescein angiography complication survey. Ophthalmology.

[3] Kwiterovich KA, Maguire MG, Murphy RP, Schachat AP, Bressler NM, Bressler SB (1991). Frequency of adverse systemic reactions after fluorescein angiography. Results of a prospective study. Ophthalmology.

[4] Izhaky D, Nelson DA, Burgansky-Eliash Z, Grinvald A (2009). Functional imaging using the retinal function imager: direct imaging of blood velocity, achieving fluorescein angiography-like images without any contrast agent, qualitative oximetry, and functional metabolic signals. Jpn J Ophthalmol.

[5] Ganekal S (2013). Retinal functional imager (RFI): non-invasive functional imaging of the retina. Nepal J Ophthalmol.

[6] Nelson DA, Krupsky S, Pollack A, Aloni E, Belkin M, Vanzetta I (2005). Special report: noninvasive multi-parameter functional optical imaging of the eye. Ophthalmic Surg Lasers Imaging.

[7] Witkin AJ, Alshareef RA, Rezeq SS, Sampat KM, Chhablani J, Bartsch DU (2012). Comparative analysis of the retinal microvasculature visualized with fluorescein angiography and the retinal function imager. Am J Ophthalmol.

[8] Nelson DA, Burgansky-Eliash Z, Barash H, Loewenstein A, Barak A, Bartov E (2011). High-resolution wide-field imaging of perfused capillaries without the use of contrast agent. Clin Ophthalmol.

[9] Chhablani J, Bartsch DU, Cheng L, Gomez L, Alshareef RA, Rezeq SS (2013). Segmental reproducibility of retinal blood flow velocity measurements using retinal function imager. Graefes Arch Clin Exp Ophthalmol.

[10] Wang L, Jiang H, Grinvald A, Jayadev C, Wang J (2018). A Mini Review of Clinical and Research Applications of the Retinal Function Imager. Curr Eye Res.

[11] Burgansky-Eliash Z, Nelson DA, Bar-Tal OP, Lowenstein A, Grinvald A, Barak A (2010). Reduced retinal blood flow velocity in diabetic retinopathy. Retina.

[12] Burgansky-Eliash Z, Barak A, Barash H, Nelson DA, Pupko O, Lowenstein A (2012). Increased retinal blood flow velocity in patients with early diabetes mellitus. Retina.

[13] Arbel Y, Sternfeld A, Barak A, Burgansky-Eliash Z, Halkin A, Berliner S (2014). Inverse correlation between coronary and retinal blood flows in patients with normal coronary arteries and slow coronary blood flow. Atherosclerosis.

[14] Birger Y, Blumenfeld O, Bartov E, Burgansky-Eliash Z (2011). Reduced retinal blood flow-velocity in severe hyperlipidemia measured by the retinal function imager. Graefes Arch Clin Exp Ophthalmol.

[15] Gutfreund S, Izkhakov E, Pokroy R, Yaron M, Yeshua H, Burgansky-Eliash Z (2013). Retinal blood flow velocity in metabolic syndrome. Graefes Arch Clin Exp Ophthalmol.

[16] Burgansky-Eliash Z, Barash H, Nelson D, Grinvald A, Sorkin A, Loewenstein A (2014). Retinal blood flow velocity in patients with age-related macular degeneration. Curr Eye Res.

[17] Barak A, Burgansky-Eliash Z, Barash H, Nelson DA, Grinvald A, Loewenstein A (2012). The effect of intravitreal bevacizumab (Avastin) injection on retinal blood flow velocity in patients with choroidal neovascularization. Eur J Ophthalmol.

[18] Böhni SC, Howell JP, Bittner M, Faes L, Bachmann LM, Thiel MA (2015). Blood flow velocity measured using the Retinal Function Imager predicts successful ranibizumab treatment in neovascular age-related macular degeneration: early prospective cohort study. Eye (Lond).

[19] Linsenmeier RA, Zhang HF (2017). Retinal oxygen: from animals to humans. Prog Retin Eye Res.

[20] Spaide RF, Klancnik JM, Cooney MJ (2015). Retinal vascular layers imaged by fluorescein angiography and optical coherence tomography angiography. JAMA Ophthalmol.

[21] Liu G, Yang J, Wang J, Li Y, Zang P, Jia Y (2017). Extended axial imaging range, widefield swept source optical coherence tomography angiography. J Biophotonics.

